# The Coronary Collateral Circulation – Past, Present and Future

**DOI:** 10.2174/1573403X113099990004

**Published:** 2014-02

**Authors:** Pascal Meier, Christian Seiler

The clinical relevance of the coronary arteries has been for decades. The evidence indicating a benefit regarding clinical outcomes including overall survival is increasing [1, 2]. Furthermore, our understanding of potential mechanism of protection is improving. While myocardial ischemia leads to electrical alterations (QT interval prolongation) which possibly put a patient at risk for fatal arrhythmias, a well-developed collateral circulation can counterbalance this effect [3, 4].

In this focus issue world-renowned experts in the field provide a very comprehensive overview of the coronary collateral circulation, the authors explore the exact clinical role of collaterals, determinants of collaterals, the mechanism of collateral growth (arteriogenesis) and therapeutic options to promote arteriogenesis. 

This focus issue will address very important questions such as: are coronary collaterals growing de-novo or are they pre-existent? What is the stimulus for collateral growth? Is it driven by ischemia? Even more importantly, can we therapeutically induce the growth of collateral vessels? 

While initial attempts to induce collateral growth have been rather unsuccessful, recent pilot studies have shown promising results which will be discussed in detail [5, 6]. These treatments originate in the improved understanding of arteriogenesis: the role of monocytes and the role of endothelial shear stress [7]. Growth factors, such as the granulocyte colony stimulating factor (G-CSF) can release monocytes from the bone marrow, external counterpulsation or physical exercise increases endothelial shear stress. All three interventions have lead to an improvement of the collateral function and they may represent treatment options for patients with severe coronary artery disease in the future [8, 9]. 

Genetic predispositions leading to heterogeneity in the collateral anastomoses has been described and genes which influence the arteriogenesis pathway have been defined [3, 10]. Future efforts have to focus on finding substances to influence these pathways by either blocking or stimulating these genes. However, we have to be aware of the risk of induction of vessel growth, including potential cancerogenic and pro-atherogenic effects.

This review series will not uncritically praise the value of the coronary collateral circulation, it will also discuss data which may indicate an adverse effect of collaterals, such as the observation that patients with extensive collaterals have a higher risk for re-stenosis after a percutaneous coronary intervention [11]. The mechanisms for this phenomenon are unclear. Potentially, competitive flow alters the shear stress on endothelial cells. An alternative explanation is the fact that collaterals simply represent a marker of more advanced disease, and are therefore associated with an increased restenosis risk.

This review series aims to improve the understanding of this increasingly important field and will provide a balanced view from a panel of pioneering experts. We will cover historical aspects, critically evaluate current evidence and also discuss future directions of investigating the coronary collateral circulation.

## COMPETING INTERESTS

The authors declare that they have no competing interests with regard to this paper.
